# Lamellar Ichthyosis with Rickets

**DOI:** 10.12669/pjms.292.3298

**Published:** 2013-04

**Authors:** Raafia Ali, Shahbaz Aman, Muhammad Nadeem

**Affiliations:** 1 Raafia Ali, Department of Dermatology, King Edward Medical University/ Mayo Hospital, Lahore, Pakistan.; 2 Shahbaz Aman, Department of Dermatology, King Edward Medical University/ Mayo Hospital, Lahore, Pakistan.; 3 Muhammad Nadeem, Department of Dermatology, King Edward Medical University/ Mayo Hospital, Lahore, Pakistan.

**Keywords:** Lamellar ichthyosis, Rickets, Vitamin D deficiency

## Abstract

Lamellar ichthyosis (LI) is a rare genetic disorder with autosomal recessive inheritance. It is equally seen in both sexes and usually manifests at birth. The child presents as a collodion baby. The erythema is minimal or absent; but when present, it is maximum on the face. The scaling is generalized, accentuated on lower extremities and flexural areas. Rickets is a condition in which there is softening of bones leading to fractures and deformities. It is caused by vitamin D deficiency & lack of adequate calcium in diet. Children, 6 to 24 months of age, are at a higher risk due to rapidly growing bones. The association between various types of ichthyoses and rickets is well documented. We report a case of lamellar ichthyosis with rickets in a 14-year-old girl from our part of the world.

## INTRODUCTION

Lamellar ichthyosis (LI) may be caused by mutations affecting different genes.^1^ There is abnormality of gene encoding for transglutaminase 1, an important epidermal enzyme responsible for cross-linking epidermal proteins during the formation and maturation of the stratum corneum.^1^^,^^2^ The condition usually presents at birth and the baby is found to be ensheathed in a membrane.^3^^,^^4^ When the membrane sheds, typical scales become visible which are broad, dark-brown, thick, adherent and plate-like in nature.^3^^,^^4^ The associated features are ectropion, scarring alopecia and palmoplantar keratoderma.^4^ Diagnosis of LI is based on the history of collodion membrane at birth and the characteristic appearance of scales, especially on the shins. Treatment includes hydration, lubrication, keratolytic application and oral acitretin.^2^^,^^4^

The Word **“**Rickets**”** is derived from ‘*Wrickken*’ meaning to twist.^5^ It occurs due to vitamin D deficiency and decreased intake of calcium in diet leading to softening of bones resulting in fractures & deformities.^6^ There may be bone pain, muscle weakness, growth disturbance, bowlegs, pigeon chest and knock-knees.^6^ Widening of wrist raises an early suspicion of this condition which is caused by the hyperplasia of metaphyseal cartilage.^6^ Treatment includes an increased dietary intake of calcium & vitamin D and/ or exposure to sunlight.^6^

In most of the developing countries, nutritional rickets is still a major health problem.^7^ There are many cases of different kinds of ichthyoses with rickets which have already been reported worldwide. To the best of our knowledge, this is the first case of lamellar ichthyosis with rickets to be reported from Pakistan.

## CASE REPORT

A 14-year-old girl resident of District Laiyyah, Pakistan, presented at the Department of Dermatology, King Edward Medical University/Mayo Hospital, Lahore, with complaints of generalized fish-like scaling since birth and bowing of legs for the last five years. Her mother reported that the child was born as a result of a full-term spontaneous vaginal delivery. At birth, her body was completely encased in a shiny taut membrane. The membrane shed over a period of one month followed by gradual development of generalized dryness and scaling of skin. After one month, thick, dark-brown, large and dry scales were visible all over her body. There was history of pain in both ankle & knee joints for the last six years and in shoulder joints for one year. The pain was aggravated by movement and relieved by rest & medication. There was no complaint of redness, swelling or stiffness of joints. She gradually developed deformities and bending of her legs over the last five years followed by difficulty in walking over the past three years. She had no history of blisters, peeling of skin, mucosal & nail involvement and photosensitivity. Systemic enquiry was insignificant. The child had undergone appropriate immunization and her dietary intake was satisfactory. There was no history of malabsorption or renal disease but history of decreased exposure to sun was present. A history of parental consanguinity was also present. Her two brothers & one sister had a similar kind of scaling on their bodies but without bony deformities while no other sibling or parents had any history of bone or skin disease. Her milestones were normal.

**Fig.1:**
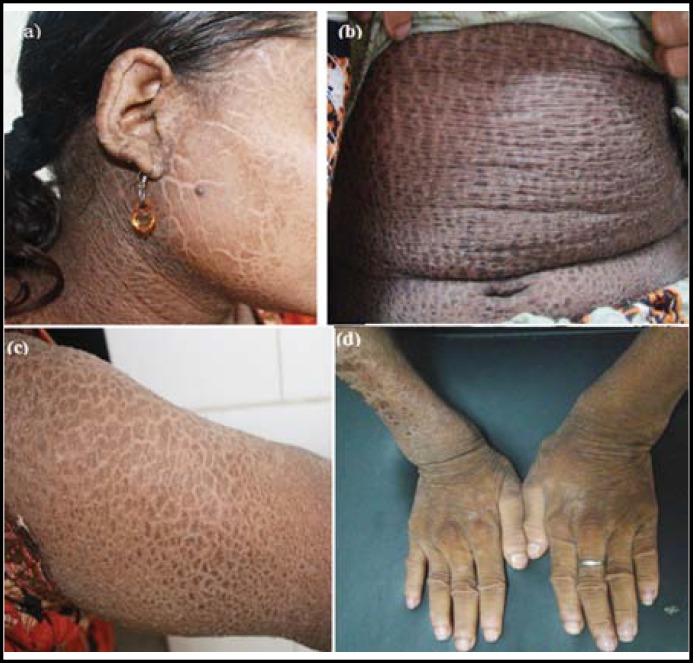
(a, b, c) Dark brown, adherent scales in lamellar pattern. (d) Widening of wrists

**Fig.2:**
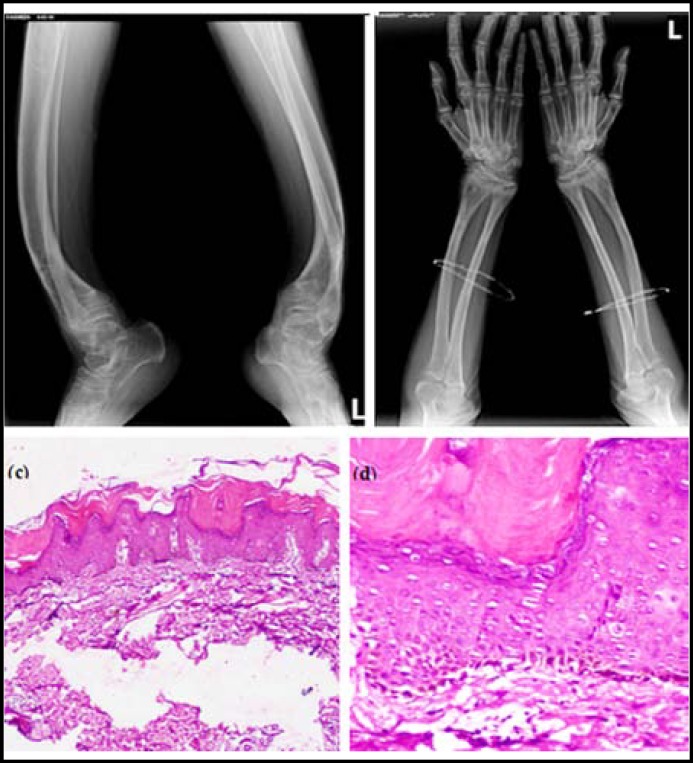
(a) Green-stick fracture. (b) Decreased bone density. (c, d) Hyperkeratosis, acanthosis, hypergranulosis, large papillae, blunt rete ridges and supra-papillary perivascular lymphocytic infiltration; HE (100x), (200x).

Examination revealed generalized dryness and scaling all over her body. The scales were dark-brown in colour, polygonal in shape, large in size, odourless, thick & adherent to skin and arranged in a mosaic pattern (Fig.1). The hyperkeratosis was present on palms & soles. Finger nails were shiny due to itching**.** There were fine scales on scalp but no ectropion on eye examination. Her mucosae, teeth and hair were normal on examination. She had signs of rickets in the form of pigeon-shaped chest, widening of the wrists & ankles and knock-knees deformity. Her lower limb bones showed marked lateral bowing (Fig.2). Both fontanelle were closed. No abnormality was detected regarding systemic examination.

Routine investigations of the blood, urine & stool revealed no abnormality. The serum calcium (6.6 mg/dl), 25-hydroxy-vitamin D (7.8 ng/ml) & 24-hour urinary calcium (1.05 mmol/24 hours) levels were low, but the serum alkaline phosphatase (1192 IU/L) and parathormone levels (354 pg/ml) were raised. Dual energy X-ray absorptiometry (DEXA) scan showed markedly reduced Bone Mineral Density. Radionuclide skeletal scintigraphy revealed expansion of multiple epiphyseal plates & adjacent bones with abnormally increased non-homogeneous uptake of radionuclide and deformity of long bones of both upper & lower limbs. There was a generalized decrease in bone density especially of long bones. Long bone deformities with bowing of the tibia, fibula, femur and radius were seen radiologically (Fig.2). Knee and wrist joints showed expansion of metaphyses & epiphyses. There was also minimal cupping along with some sclerotic changes at the wrist joint which indicates an evidence of healing changes of rickets (Fig.2). There was an incomplete fracture with marginal sclerosis at the meta-diaphysial end of fibula called green-stick fracture which is common in rickets (Fig.2). Ultrasonography of abdomen and pelvis showed no abnormality. Skin biopsy revealed hyperkeratosis, acanthosis, hypergranulosis, large papillae with blunt rete ridges and a perivascular lymphocytic infiltration in the supra-papillary region of dermis (Fig.2).

On the basis of these findings, a diagnosis of lamellar ichthyosis with rickets was made and the child was treated with parenteral vitamin D3 (600,000 units, intramuscular monthly) and oral calcium (1000 mg daily in two divided doses) with vitamin D supplements. Regular sun exposure and a diet rich in vitamin D & calcium was advised. Emollients and keratolytics were applied for her skin condition. In a follow-up period of five months, we noticed some clinical, radiological and serological improvement. We are now planning for orthopedic surgery (osteotomy) for correction of bony deformities and systemic retinoids for further improvement of skin condition.

## DISCUSSION

The word ichthyosis is derived from the Greek **‘*ichthys’*** meaning* ‘*Fish’ due to the similarity in appearance to fish-like scales.^2^ Lamellar ichthyosis is an autosomal recessive disorder that is apparent at birth and persists throughout life.^1^ There is minimal erythema while the scales are large, thick, dark- brown in colour and firmly adherent.^1^^,^^2^ Sweating is also impaired.^1^^,^^2^ Majority of cases with rickets are found among children.^8^ It develops in a child when he or she lacks vitamin D, phosphate, or calcium in diet.^7^^,^^8^ Children that are kept away from the sun can develop rickets as skin has also the ability to synthesize vitamin D, as long as it is exposed to the ultraviolet radiation from sunlight.^7^^,^^8^

In our patient, the diagnosis of lamellar ichthyosis with rickets was made because of the clinical, histopathological, biochemical and radiological findings. The condition has to be differentiated from non-bullous ichthyosiform erythroderma, X-linked ichthyosis, Chanarin-Dorfman syndrome and Trichothiodystrophy.^2^^,^^4^

In patients of non-bullous ichthyosiform erythroderma, the scales are thin, superficial, white or grey & semi adherent in nature, which were not seen in our patient.^2^ The scales in X-linked Ichthyosis are almost similar to lamellar ichthyosis but these are medium in size, light in colour and the presence of extra-cutaneous manifestations, obstetric complications & male gender helped to delineate the condition.^2^^,^^9^

Chanarin-Dorfman syndrome is a rare, recessively inherited, lipid storage disorder.^2^ The scales are usually fine and white on an erythrodermic background.^2^ Lamellar ichthyosis-like scales may be present on legs & trunk but the differentiating features are leukocyte vacuoles in peripheral blood picture, hepatosplenomegaly and involvement of muscles & central nervous system, due to errors of triacylglycerol metabolism.^2 ^The patient may have cataract and diffuse erythroderma.^2^

Trichothiodystrophy also denoted as IBIDS (ichthyosis, brittle hair, impaired intelligence, decreased fertility, short stature) is a condition in which scales vary from fine to erythrodermic in nature.^2^^,^^4^ Additional features like eczema, flexion contractures, nail dystrophy, elfin-like progeric face, fat atrophy, prominent ears & chin recession^2^ present in this condition were absent in our patient.

In our case, vitamin D-deficient rickets is most likely due to a combination of sun avoidance, poor penetration of skin by sunlight (increased keratinocyte proliferation) and impaired skin production of vitamin D. A low serum 25-hydroxyvitamin D_3_ level in the absence of other causes of vitamin D deficiency supported our diagnosis. This is in accordance with the literature that suggests different factors for development of rickets in skin diseases;^10^ (i) alterations in epidermal cholesterol metabolism possibly involving vitamin D receptors, (ii) increased keratinocyte proliferation resulting in poor or no penetration of skin by sunlight, (iii) associated vitamin D-dependent rickets and (iv) limited sun exposure to prevent sunburn and sunstroke.^10^
